# Cinnamaldehyde Inhibits the Replication of Porcine Reproductive and Respiratory Syndrome Virus Type 2 In Vitro

**DOI:** 10.3390/v17040506

**Published:** 2025-03-31

**Authors:** Junzhu Song, Jingyu Zhang, Jian Chen, Songbiao Chen, Zuhua Yu, Lei He, Ke Ding, Ying Wei

**Affiliations:** 1 Laboratory of Functional Microbiology and Animal Health, College of Animal Science and Technology, Henan University of Science and Technology, Luoyang 471003, Chinayzhd05@163.com (Z.Y.);; 2 Luoyang Key Laboratory of Live Carrier Biomaterial and Animal Disease Prevention and Control, Luoyang 471003, China; 3 The Key Laboratory of Animal Disease and Public Health, Henan University of Science and Technology, Luoyang 471023, China; 4 College of Animal Science and Veterinary Medicine, Henan Institute of Science and Technology, Xinxiang 453003, China

**Keywords:** PRRSV, cinnamaldehyde, antiviral activity, dsRNA

## Abstract

Globally, the swine industry suffers significant economic losses due to the presence of porcine reproductive and respiratory syndrome virus (PRRSV). Unfortunately, existing vaccines fail to offer adequate protection against the various strains of PRRSV, and there are currently no specific treatments available for this virus. In this study, we screened four natural products and identified cinnamaldehyde (CA) as an effective inhibitor of PRRSV infection in Marc-145 cells. CA could achieve an inhibition rate of up to 93% on PRRSV N protein at 160 μM. Mechanistically, CA exerted anti-PRRSV ability in different treatment modes. CA could directly interact with PRRSV particles. Cinnamaldehyde blocks the binding, entry, replication, and release of PRRSV. Furthermore, a significant reduction in dsRNA levels was observed in the CA-treated groups compared to the control groups. In conclusion, our research demonstrated that CA could inhibit essential stages of the PRRSV lifecycle: binding, entry, replication, and release. CA could directly interact with PRRSV. Additionally, CA disrupted the expression of dsRNA during viral replication, thereby suppressing in vitro PRRSV replication in Marc-145 cells. This study provides crucial perspectives on the potential application of CA for the prevention and treatment of PRRS.

## 1. Introduction

Porcine reproductive and respiratory syndrome (PRRS) is a highly infectious and immune-compromising disease resulting from PRRSV. Symptoms include reproductive issues in sows and respiratory problems in piglets, causing significant global economic losses [1]. The virus is an enveloped, single-stranded RNA virus with positive polarity, having a genome around 15 kb in size. It contains at least 10 open reading frames (ORFs) that code for proteins essential for viral replication and structure. The viral replicase genes, encompassing ORF 1a and ORF 1b, collectively encode at least 14 functional nonstructural proteins (NSPs) which are vital in viral replication, modulation of virulence, and evasion of host immune response [2,3,4]. Replication and transcription complexes (RTCs) primarily consist of networks formed by viral replicase proteins along with certain cellular counterparts [5,6]. Proper assembly of the RTC is crucial for efficient synthesis of viral RNA while coordinating the intricate processes of transcription and replication [7]. Currently, vaccination remains the cornerstone for prevention and control of PRRS [8]. However, continuous mutation-driven antigenic variation poses challenges to the efficacy of traditional vaccines against emerging heterologous strains [9,10,11,12,13]. Moreover, excessive use of attenuated vaccines carries safety risks such as virulence reversion and recombination events that augment PRRSV infection rates in pigs while driving further strain diversification through mutational accumulation, creating a detrimental cycle [14,15]. The prevention and control of PRRS remains a serious issue, and searching for new anti-PRRSV drug targets will emerge as a new approach for future prevention and control strategies [5,16].

Natural products have always been a pivotal source of potential lead drugs, and their structural analogues have historically made significant contributions to the treatment of viral diseases. In comparison to conventional chemosynthetic molecules, natural products exhibit remarkable advantages in new drug discovery owing to their diverse skeletal structures and intricate complexities. Research has revealed that various natural products possess antiviral properties against PRRSV [17]. For instance, emodin activates Toll-like receptor 3 to inhibit PRRSV [18]; proanthocyanidin A2 directly interacts with PRRSV [19]; epigallocatechin gallate hampers PRRSV replication by interfering with lipid metabolism [20]; toosendanin inhibits PRRSV through caspase-1 activation and IL-1β maturation via an IFI 16-dependent pathway [21]; tubercidin impedes PRRSV replication via RIG-I/NF-κB pathways while disrupting viral NS2 synthesis [22]. However, there is currently an absence of commercially available antiviral drugs specifically targeting PRRSV. Paeonol, a phenolic substance predominantly extracted from the root bark and/or cortex of plants belonging to the genus Paeonia, particularly peonies and Chinese herbaceous peonies, has demonstrated significant inhibitory effects against human respiratory syncytial virus, influenza virus, and hepatitis B virus [23,24,25]. The root of *Lobed kudzuvine* is often employed in conventional Chinese medicine as a preventive measure against fever, diarrhea, and inflammatory diseases [26]. Puerarin, the principal bioactive constituent found in the root of *Lobed kudzuvine*, possesses considerable nutritional and health benefits. It has demonstrated inhibitory effects on influenza A virus [27,28,29]. Luteolin, a flavonoid compound present in numerous medicinal plants, has been reported to exhibit inhibitory activity against multiple viruses, including Japanese encephalitis virus, pseudorabies virus, etc. [30,31,32,33,34]. *Cinnamomum tamala* is extensively utilized across Asia for both culinary and medicinal purposes due to its high content of cinnamaldehyde (CA) content, which serves as its primary active component [35]. Research has indicated that CA has a diverse range of beneficial effects, including antibacterial, anticancer, and antiviral properties [36,37,38]. Notably, CA has shown substantial antiviral activity against multiple viruses like influenza A/PR/8 virus [39], coxsackievirus B3 [40], and viral myocarditis [41].

In the present study, we screened four natural products and identified CA as an effective inhibitor of PRRSV infection in Marc-145 cells at submicromolar concentrations in a dose-dependent manner. Mechanistically, CA is capable of direct interaction with PRRSV particles. CA targeted the binding, entry, replication, and release stages of the virus while impeding viral RNA synthesis. This study provides crucial perspectives on the potential application of CA for the prevention and treatment of PRRS.

## 2. Materials and Methods

### 2.1. Cells and Virus

Marc-145 cells, which are kidney cells originating from African green monkeys, were cultivated in Dulbecco’s Modified Eagle’s Medium (DMEM) sourced from CORNING, Suzhou, China. The medium was enriched with a 10% concentration of fetal bovine serum (FBS) provided by EVERY GREEN, Shanghai, China. The cells were then incubated at a temperature of 37 °C in an environment containing 5% CO_2_. The highly pathogenic PRRSV strain Li11 (preserved in our laboratory) was propagated and titrated using Marc-145 cells [42]. Porcine alveolar macrophages (PAMs) were harvested from the lungs of 4- to 6-week-old Large-White piglets, which were confirmed to be PRRSV-negative, using a lung lavage technique based on a method previously detailed [43]. The study protocol was approved by the Ethics Committee of Henan University of Science and Technology, China Approval Number HAUST-024-PI1203008 (12 May 2024). In summary, the lung tissues underwent three rounds of washing with a precooled phosphate-buffered saline (PBS) solution that included 300 IU/mL of penicillin and 300 g/mL of streptomycin. Following this, the cells were subjected to centrifugation at 800× *g* for a duration of 10 min. Subsequently, the cells were resuspended in RPMI 1640 medium, which was enhanced with 10% fetal bovine serum (FBS), 100 IU/mL of penicillin, and 100 g/mL of streptomycin. The cell suspension was adjusted to a concentration of 1 × 10^6^ cells/mL within a 6-well plate setup. Finally, the cells were incubated at a temperature of 37 °C for a period of 6 h.

### 2.2. Preparation of Compounds

Cinnamaldehyde, puerarin, paeonol, and luteolin were purchased from MedChemExpress (Monmouth Junction, South Brunswick, NJ, USA) with purities of 99.1%, 99.42%, 99.98%, and 98.72%, respectively. IFN-α, a broad-spectrum antiviral drug obtained from Sangon Biotech (Shanghai, China), served as the positive control [44]. Cinnamaldehyde, puerarin, paeonol, and luteolin were dissolved in dimethyl sulfoxide (DMSO, Solarbio, Beijing, China) and subsequently diluted using a medium containing 2% FBS to achieve the desired storage concentration for each compound individually. Interferon-α (IFN-α) was diluted using distilled water to attain the desired storage concentration. Prior to usage, all compounds were further diluted to their respective working concentrations using a medium comprising 2% FBS solution. The final content of DMSO during application remained below 0.1%.

### 2.3. Cell Viability Assay

Marc-145 cells or PAMs were grown in 96-well plates until reaching 80% confluency. Then, CA, puerarin, paeonol, and luteolin were added for a duration of 24 h. Cell viability was assessed using the Cell Counting Kit 8 (CCK-8) from Beyotime, China, adhering to the manufacturer’s guidelines. Each well was then treated with 10 μL of CCK-8 solution in the dark and incubated for one hour at 37 °C in an environment containing 5% CO_2_. The absorbance at a wavelength of 450 nm was subsequently measured [22].

### 2.4. Antiviral Activity Assays

An experiment to evaluate the antiviral effects and compare the inhibitory abilities of various natural compounds against PRRSV in vitro was conducted. Marc-145 cells or PAMs were grown in 24-well plates until achieving 80% confluency, followed by the addition of CA, purarin, paeonol, and luteolin into the wells for a 1 h incubation period. After three washes with PBS, PRRSV (MOI = 1) was added and incubated for another hour. Subsequently, the viral solution was removed and the wells were rinsed another three times with PBS. The four natural compounds were then reintroduced and incubated at 37 °C with 5% CO_2_ for a duration of 24 h. The antiviral effect was evaluated using indirect immunofluorescence assay (IFA) experiments.

The antiviral detection of CA was selected for further investigation, and the sample treatment method remained consistent with the abovementioned procedure. Cells and supernatants were separately collected at 24 h. To maximize the release of cellular virions, cells and supernatants underwent three freeze–thaw cycles at temperatures of 80 °C as well as 4 °C. The final viral titer in the supernatant was determined using a TCID_50_ assay, while the viral N gene mRNA level was evaluated with real-time quantitative reverse-transcription PCR (RT-qPCR). PRRSV-infected cell count was assessed by the IFA, whereas the viral N protein level was analyzed through Western blotting. IFN-α was utilized as a benchmark for assessing antiviral drug efficacy.

### 2.5. Time-of-Addition Assay

Marc-145 cells were grown in 24-well plates until reaching confluence and then infected with PRRSV for a duration of 2 h at 37 °C. CA was administered either before (pre-treatment), during (co-treatment), or after (post-treatment) PRRSV infection. For pre-treatment, the cells were incubated with CA for 2 h, 4 h, 6 h, or 8 h at 37 °C [45]. Following this, the cells were washed three times with PBS. Subsequently, the cells were infected with PRRSV for 2 h. In the case of co-treatment, the cells were exposed to PRRSV at a temperature of 37 °C simultaneously. After an incubation period of 2 h, the mixture was discarded and the cells were washed three times with PBS. Fresh medium was then added to the wells. Under post-treatment, the cells were initially infected with PRRSV for 2 h at a temperature of 37 °C and subsequently incubated in fresh medium containing CA for durations of either 2 h, 4 h, 6 h, or 8 h. This was followed by incubation in fresh medium. At 24 h post-infection (hpi), the TCID_50_ assay as well as RT-qPCR and IFA analyses were performed.

### 2.6. Direct PRRSV-CA Interaction

To explore the possibility of direct interaction between CA and the virus, 100 microliters of PRRSV at an MOI of 1 was combined with varying concentrations of CA in essential medium (total volume of 0.9 mL). This mixture was then incubated for one hour at a temperature of 37 °C [46]. Subsequently, PRRSV and CA were separated using ultrafiltration centrifugation. The mixture containing PRRSV and the compounds was transferred to an ultrafiltration device with a 0.5 mL capacity and a molecular weight cutoff of 30 kilodaltons. The mixture was centrifuged at 7500 times gravity for 10 min at a temperature of 4 degrees Celsius. The PRRSV particles trapped in the ultrafiltration tube were washed three times with essential medium to eliminate any residual CA. After washing, the PRRSV particles were resuspended in essential medium and used to infect Marc-145 cells that had been grown in 24-well plates for two hours. Following three washes with PBS, the infected cells were cultured in fresh medium for an additional 24 h at 37 °C. The expression of viral mRNA and N protein was then determined using qRT-PCR and the IFA.

### 2.7. Viral Binding, Entry, Replication, and Release Assays

Viral binding, entry, replication, and release assays were conducted as described in a previous study [47].

In the binding assay, Marc-145 cells were chilled at 4 °C for a duration of 30 min and then subjected to infection with a PRRSV-CA mixture at the same low temperature for 2 h. Following this, the supernatant was removed, and the cells were rinsed three times using cold PBS. The cells were then harvested to isolate total RNA for further analysis.

For the entry experiment, Marc-145 cells were cooled to 4 °C for 30 min before being infected with PRRSV under the same conditions for 2 h. After rinsing with cold PBS, the cells were exposed to CA at a temperature of 37 °C for 2 h. Subsequently, the cells were washed with PBS and gathered to assess PRRSV mRNA expression through RT-qPCR.

In the replication assay, Marc-145 cells were initially infected with PRRSV for 4 h, encompassing both binding and entry phases, and then treated with CA. Ultimately, cell samples were collected at various time points—6, 12, 24, 36, and 48 h post-infection (hpi)—to evaluate PRRSV mRNA expression via RT-qPCR.

To conduct the release assay, Marc-145 cells were infected with PRRSV for 24 h and the supernatant was removed. CA was then added to the cells at 8 h at a temperature of 37 °C for an additional period of time, after which the supernatant was collected for TCID_50_ determination.

### 2.8. dsRNA Assay

Marc-145 cells were inoculated in 24-well plates and subsequently subjected to PRRSV for 2 h. Following this, the supernatant was removed, and the cells underwent three washes with PBS. Samples were collected at both 6 h and 24 hpi, respectively, in the presence or absence of CA for subsequent IFA detection.

### 2.9. Indirect Immunofluorescence Assay (IFA)

Viral replication was detected using an indirect immunofluorescence assay. Marc-145 cells or PAMs were grown in 24-well plates and exposed to CA as well as PRRSV at specified time points. The treated specimens underwent three washes with PBS, followed by fixation in 4% paraformaldehyde at RT for a duration of 15 min. Subsequently, the samples were permeabilized using 0.25% Triton X-100 (Solarbio, Beijing, China) in PBS at RT for another 15 min. After a final wash with PBS, the Marc-145 cells were incubated in a 4% solution of bovine serum albumin (BSA, sourced from BBI, Beijing, China) at RT for one hour to inhibit non-specific binding sites. Subsequently, they underwent three washes with PBST and were incubated either with anti-PRRSV N mAb (JNT) or anti-dsRNA antibody (Mouse anti-double-stranded RNA [J2], Nordic MUbio, Susteren, The Netherlands) at 37 °C for 1 h. Following another round of washing with PBST three times, the cells were further incubated with CY3-labeled goat anti-mouse IgG (ELENCE) at 37 °C for one hour to detect the bound antibodies. Finally, nucleus staining was performed using DAPI (Beyotime, Beijing, China), and inverted fluorescence microscopy (ZEN, Düsseldorf, Germany) was used to visualize the results.

### 2.10. Western Blotting

Marc-145 cells cultured in 6-well plates were harvested using lysis buffer (Beyotime, China) and separated by 12% SDS-PAGE. Proteins were then transferred onto polyvinylidene difluoride (PVDF) membranes (Millipore, St. Louis, MO, USA). Following blocking with 4% bovine serum albumin (BSA) for one hour at RT, they were incubated with anti-PRRSV N antibody (JNT) and GAPDH mouse mAb (Proteintech, Rosemont, IL, USA), as indicated in the figures. After three washes with TBS-Tween, membranes were further incubated with HRP-conjugated secondary antibodies (Proteintech, USA) for 1 h at RT. Protein bands were visualized using ECL Plus chemiluminescence reagent (Beyotime, China).

### 2.11. Real-Time Quantitative PCR (RT-qPCR)

The FastPure Cell/Tissue Total RNA Isolation KIT v2 (Vazyme, Nanjing, China) was utilized to isolate total RNA from Marc-145 cells or PAMs. Subsequently, reverse transcription was conducted with the HiScript^®^ⅢRT SuperMix for qPCR (+gDNA wiper) Vazyme Code: R323-01 100 rxns (Vazyme, China), based on the manufacturer’s instructions. RT-qPCR analysis was conducted with the ChamQ Universal SYBR qPCR Master Mix (Vazyme, China) on a Light-Cycler 480 PCR system (Roche, Rotkreuz, Switzerland), as described previously [48,49]. The primers employed in this study were as follows: PRRSV-N-F, 5′-TAAGATCATCGCCCAACAAA-3′; PRRSV-N-R, 5′-TCGGCAAACTAAACTCCACA-3′; mGAPDH-F, 5′-GGGAGCCAAAAGGGTCATCA-3′; mGAPDH-R, 5′-CGTGGACTGTGGTCATGAGT-3′; pGAPDH-F, 5′-GATGCTGGTGCTGAGTATGT-3′; pGAPDH-R, 5′-GGCAGAGATGATGACCCTTT-3′. Data analysis was performed using the 2^−ΔΔCT^ method.

### 2.12. TCID_50_ Assay

For PRRSV titration, Marc-145 cells or PAMs were inoculated into 96-well plates at the appropriate density. The virus supernatant was prepared by serial ten-fold dilution, and 100 μL of the diluent was added to each well in 6 replicates. The virus titer was computed at 5 dpi with the Reed–Muench method [50].

### 2.13. Statistical Analysis

The samples were assayed in triplicate, and the data are shown as the mean ± SD. Data analysis was carried out with Prism 5 software (GraphPad). The significance of differences between the treatment and control groups was determined by a two-tailed unpaired *t* test. Statistical significance was taken into consideration when the *p* value was less than 0.05 (*, *p* < 0.05; **, *p* < 0.01).

## 3. Results

### 3.1. Effect of Four Compounds on Cell Viability and Anti-PRRSV Activity

**Figure 1:**
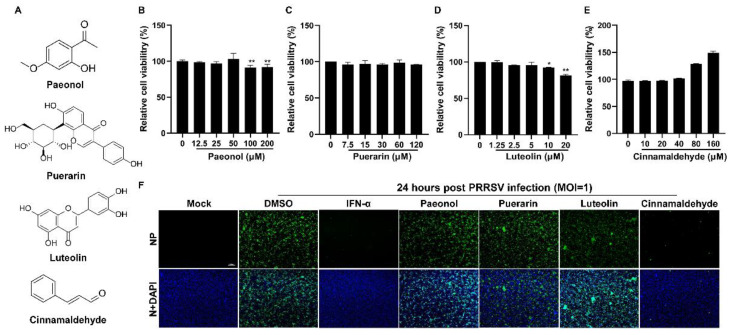
The effect of four compounds on cell viability and anti-PRRSV activity. (**A**) The chemical structures of paeonol, puerarin, luteolin, and cinnamaldehyde are depicted. (**B**–**E**) The cytotoxicity of four compounds on Marc-145 cells was evaluated using the CCK-8 assay. The non-toxic concentrations of four compounds were determined to be 50 μM (**B**), 120 μM (**C**), 5 μM (**D**), and 160 μM (**E**), respectively. (**F**) An immunofluorescence assay was used to investigate the anti-PRRSV (MOI = 1) effect of these compounds. IFN-α was used as a positive control. Bar = 100 µm. The data are the results of three independent experiments (means ± SD). Significant differences are denoted by * *p* < 0.05 and ** *p* < 0.01.

The chemical structures of paeonol, puerarin, luteolin, and CA are illustrated in Figure 1A. Initially, we evaluated the cytotoxicity of these four compounds on Marc-145 cells utilizing the CCK-8 assay. As depicted in Figure 1B–E, the non-toxic concentrations for the four compounds were determined to be 50 μM, 120 μM, 5 μM, and 160 μM, respectively. Subsequently, we investigated the antiviral efficacy of these compounds against PRRSV. As indicated in Figure 1F, CA exhibited a significant inhibitory effect on PRRSV infection in Marc-145 cells, whereas the other three compounds did not show notable antiviral activity. Therefore, we chose CA for further antiviral research.

### 3.2. Cinnamaldehyde Suppresses PRRSV Infection in Marc-145 Cells

**Figure 2:**
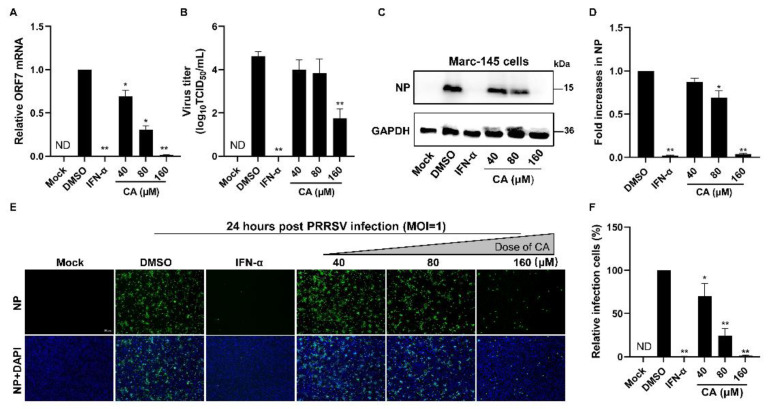
Cinnamaldehyde suppresses PRRSV infection in Marc-145 cells. (**A**,**B**) The effect of different concentrations (40, 80, 160 μM) of CA on PRRSV (MOI = 1) replication in Marc-145 cells was investigated by RT-qPCR (**A**) and a viral titer assay (**B**). (**C**–**F**) Western blotting (**C**) and an immunofluorescence assay (**E**) were used to detect the expression of PRRSV N protein at 24 hpi in the presence of CA (40, 80, 160 μM). Statistical analysis of Western blotting (**D**) and the IFA (**F**). Bar = 100 µm. The data are the results of three independent experiments (means ± SD). Significant differences are denoted by * *p* < 0.05 and ** *p* < 0.01.

Given the demonstrated notable antiviral properties of CA, our study aimed to investigate its potential antiviral activity at varying concentrations. First, we added CA into the wells for a 1 h incubation period. After three washes with PBS, PRRSV was added and incubated for another hour. Subsequently, the virus solution was removed and the wells underwent three washes with PBS. CA was next reintroduced and incubated at 37 °C with 5% CO_2_ for a duration of 24 h. To assess the impact of different CA concentrations on PRRSV replication in Marc-145 cells infected with PRRSV, we used RT-qPCR, virus titration, and Western blotting. As illustrated in Figure 2A, a dose-dependent inhibition of PRRSV proliferation was observed upon treatment with CA, with the most significant inhibitory effect manifesting at a concentration of 160 μM. Virus titration results (Figure 2B) revealed a substantial decrease in PRRSV titers following increasing doses of CA treatment. Moreover, we evaluated the inhibitory effects of three distinct concentrations of CA on viral N protein levels at 24 hpi. Our analysis showed that CA could achieve an inhibition rate of up to 93% on PRRSV N protein at 160 μM (Figure 2C–F). In conclusion, CA exerts a pronounced suppressive effect on the replication of PRRSV in Marc-145 cells through significantly reducing the virus titer, virus N protein level, and ORF7 mRNA level.

### 3.3. Cinnamaldehyde Suppresses PRRSV Infection in PAMs

**Figure 3:**
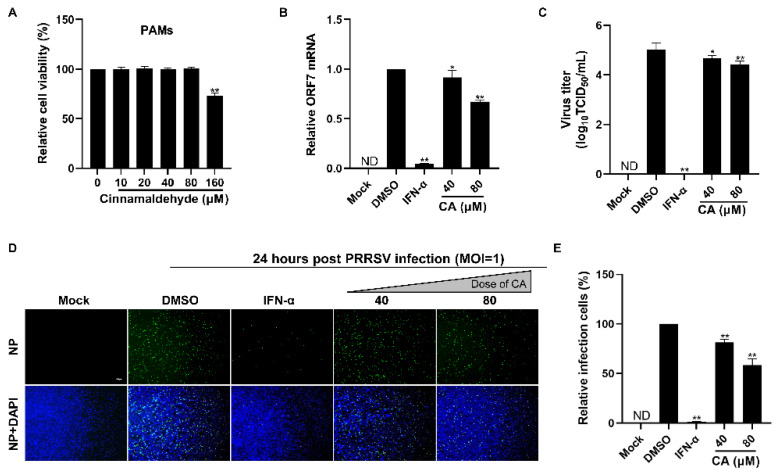
Cinnamaldehyde suppresses PRRSV infection in PAM cells. (**A**) The cytotoxicity of CA on PAM cells was evaluated using the CCK-8 assay. The non-toxic concentration of CA was determined to be 80 μM. (**B**,**C**) The effect of different concentrations (40, 80 μM) of CA on PRRSV (MOI = 1) replication in PAM cells was investigated by RT-qPCR (**B**) and a viral titer assay (**C**). (**D**,**E**) An immunofluorescence assay (**D**) was used to detect the expression of PRRSV N protein at 24 hpi in the presence of CA (40, 80 μM). Statistical analysis of the IFA (**E**). Bar = 100 µm. The data are the results of three independent experiments (means ± SD). Significant differences are denoted by * *p* < 0.05 and ** *p* < 0.01.

Given that CA demonstrated a strong antiviral effect against PRRSV infection in Marc-145 cells, we set out to determine if CA could also impede the ex vivo replication of PRRSV in PAMs, which are the primary target cells for PRRSV infection in swine. We began by evaluating the cytotoxic impact of CA on PAMs utilizing a CCK-8 assay. The results are illustrated in Figure 3A. The cytotoxic characteristics of CA against PAMs were different from those against Marc-145 cells, and we found that 160 µM of CA was cytotoxic, while 80 µm was the maximum safe concentration. Subsequently, we assessed the inhibitory impact of CA on PRRSV replication in PAMs employing RT-qPCR, virus titration, and an IFA. The findings depicted in Figure 3B–E reveal that CA notably diminished ORF7 mRNA expression, offspring virus generation, and N protein levels. The results showed that CA also had a significant inhibitory effect on PRRSV in PAM cells at 80 μM.

### 3.4. Cinnamaldehyde Exerts Anti-PRRSV Ability in Different Treatment Modes

**Figure 4:**
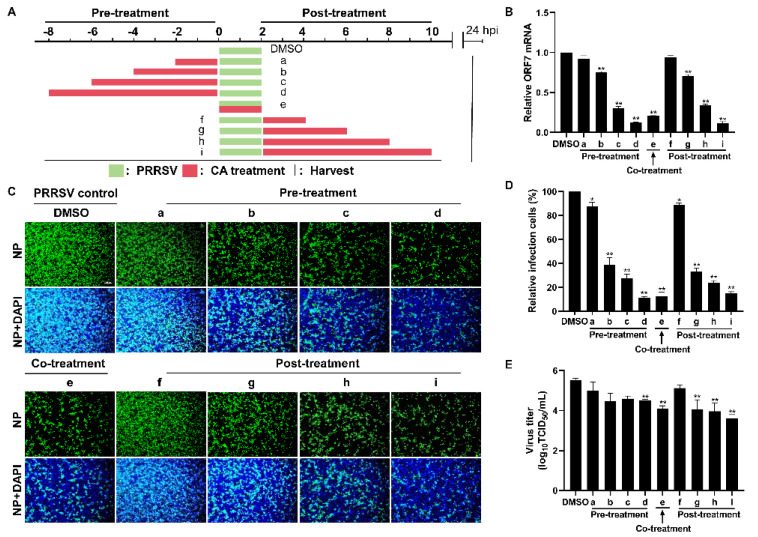
Cinnamaldehyde exerts anti-PRRSV ability in different treatment modes. (**A**) The diagram shows three different administration treatment modes. Pre-treatment: Marc-145 cells were pre-treated with CA at 160 μM at different times (2, 4, 6, 8 h) and then infected with PRRSV for another 2 h. Co-treatment: Marc-145 cells were infected with PRRSV and incubated with CA (160 μM) for 2 h. Post-treatment: Marc-145 cells were infected with PRRSV for 2 h, then treated with CA at 160 μM at different times (2, 4, 6, 8 h). Samples were harvested at 24 hpi. (**B**) RT-qPCR was used to determine viral ORF7 expression. (**C**) The expression of PRRSV N protein was detected by an IFA. (**D**) Statistical analysis of the IFA. (**E**) Cell supernatants were collected for the TCID_50_ assay. Bar = 100 µm. The data are the results of three independent experiments (means ± SD). Significant differences are denoted by * *p* < 0.05 and ** *p* < 0.01.

The time-of-addition assay was performed to investigate the specific stages of the PRRSV replication cycle at which CA exerts its antiviral effects. In this assay, cells were infected with PRRSV for a duration of 2 h, followed by the administration of CA at different incubation times: prior to infection (pre-treatment), concurrently with infection (co-treatment), and subsequent to infection (post-treatment) (Figure 4A). As illustrated in Figure 4B, neither pre-treating nor post-treating cells with CA for 2 h resulted in a reduction in viral mRNA expression. However, when cells were pre-treated or post-treated with CA for 4 to 8 h, a conspicuous reduction in viral mRNA expression was observed, which correlated directly with the passage of time. Comparable results were obtained in virus titer experiments and assessments of viral N protein levels (Figure 4C–E). Remarkably, co-treatment of PRRSV with CA also exhibited significant inhibitory effects. By measuring levels of viral mRNA, N protein, and virus titer, we demonstrated that CA could effectively inhibit the replication of PRRSV in Marc-145 cells in different treatment modes.

### 3.5. Cinnamaldehyde Directly Interacts with PRRSV

**Figure 5:**
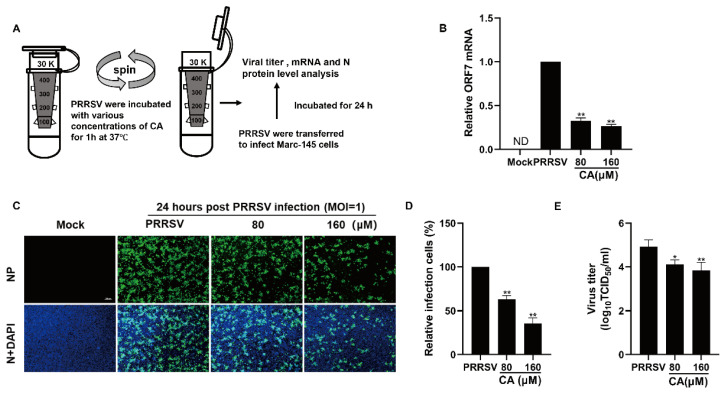
Cinnamaldehyde directly interacts with PRRSV Virions. (**A**) CA directly interacts with PRRSV virions. PRRSV was incubated with AS at various concentrations in essential medium for 1 h at 37 °C, and then PRRSV was separated from CA via ultrafiltration. (**B**–**E**) Recovered PRRSV was resuspended to infect Marc-145 cells. At 24 hpi, the cells and supernatants were harvested for determining viral mRNA using RT-PCR (**B**), viral NP detection using an IFA (**C**,**D**), and the virus titer using an endpoint dilution assay (**E**). Bar = 100 µm. The data are the results of three independent experiments (means ± SD). Significant differences are denoted by * *p* < 0.05 and ** *p* < 0.01.

As demonstrated earlier, CA displayed a strong suppressive effect on the replication of PRRSV when applied concurrently, which prompted us to explore potential direct interactions between CA and PRRSV. To address this inquiry, PRRSV was mixed with varying concentrations of CA at 37 degrees Celsius for one hour, subsequently undergoing ultrafiltration to isolate the virus from the compound. The PRRSV-containing ultrafiltrate was then reconstituted to inoculate Marc-145 cells for two hours before transferring them into fresh culture medium (Figure 5A). At 24 h post-infection, samples were gathered to assess viral N protein expression, viral load, and viral gene mRNA levels. Exposure to CA led to a significant diminution in viral mRNA quantities (Figure 5B). Furthermore, as shown in Figure 5C,D, CA treatment induced a dose-dependent reduction in viral N protein expression. Compared to DMSO-treated controls, CA treatment resulted in a greater than 65% decrease in viral N protein expression. The treatment of CA also reduced the viral titer of the progeny (Figure 5E). These results suggest that CA can interact directly with virions.

### 3.6. Cinnamaldehyde Blocks the Binding, Entry, Replication, and Release of PRRSV

**Figure 6:**
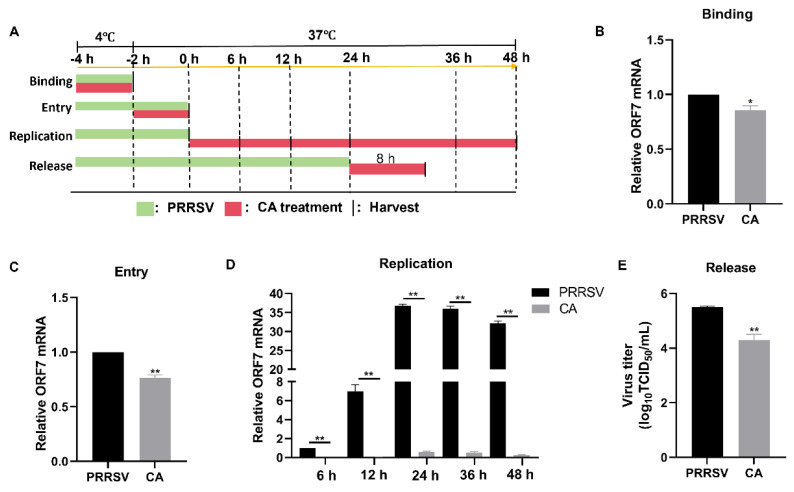
Cinnamaldehyde blocks the binding, entry, replication, and release of PRRSV. (**A**) A schematic diagram of PRRSV binding, entry, replication, and release. (**B**) A viral binding assay was performed. PRRSV was inoculated in the presence or absence of CA at 4 °C for 2 h. RT-qPCR was used to detect viral ORF7 expression. (**C**) A viral entry assay was performed. After virus binding, cells were transferred to 37 °C for another 2 h. RT-qPCR was used to analyze viral ORF7 expression. (**D**) Viral replication assays were performed. Cells were infected with PRRSV for 4 h (including binding and entry) and then treated with CA for another 6, 12, and 24 h. Cells were collected for RT-qPCR analysis. (**E**) A viral release assay was performed. Marc-145 cells were infected with PRRSV for 24 h. Then, cells were treated with CA for another 8 h. Cell supernatants were collected for the TCID_50_ assay. The data are the results of three independent experiments (means ± SD). Significant differences are denoted by * *p* < 0.05 and ** *p* < 0.01.

To investigate the mechanism of action of CA on viral proliferation, we examined the effects of CA on the lifecycle of PRRSV. The process of PRRSV infection in host cells can be segmented into four distinct stages: binding, entry, replication, and release (Figure 6A). Initially, we co-incubated PRRSV and CA at 4 °C for 2 h to facilitate viral binding without entry. Subsequently, samples were collected for RT-qPCR analysis. The results demonstrated that CA treatment did affect the levels of ORF7 mRNA, providing evidence that CA had an impact on PRRSV binding (Figure 6B). Then, Marc-145 cells were incubated with PRRSV at 4 °C for 2 h and for an additional 2 h at 37 °C in the presence or absence of CA. RT-qPCR analysis confirmed that viral entry was affected by CA (Figure 6C). We then collected samples at 6, 12, 24, 36, and 48 hpi for RT-qPCR assays. The outcomes showed that CA significantly decreased mRNA expression of ORF7 at 6, 12, 24, 36, and 48 h, indicating that CA significantly inhibited the replication phase of PRRSV. Finally, for the release assay, Marc-145 cells were infected with PRRSV (MOI = 1) for 24 h. This was followed by an additional 8 h treatment with CA, at which time the virus was released from the cells. Cell supernatants from TCID_50_ were gathered to determine progeny virus titers. The results indicated that CA blocked the binding, entry, replication, and release of PRRSV.

### 3.7. Cinnamaldehyde Inhibits the Synthesis of PRRSV dsRNA

**Figure 7:**
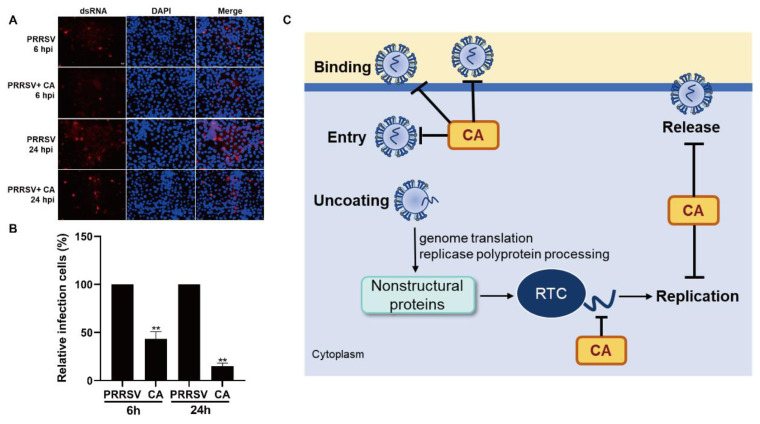
Cinnamaldehyde inhibits the synthesis of PRRSV dsRNA. (**A**,**B**) CA reduced viral dsRNA synthesis. Marc-145 cells were infected with PRRSV for 6 h or 24 h in the presence or absence of CA. Cells were fixed and stained with dsRNA antibody, followed by CY3-labeled goat anti-mouse IgG (red). Nuclei were counterstained with DAPI (blue). Bar = 50 µm. (**C**) A schematic model of the inhibition of PRRSV by CA. CA effectively inhibited PRRSV replication and release in Marc-145 cells by inhibiting viral dsRNA synthesis. The data are the results of three independent experiments (means ± SD). Significant differences are denoted by ** *p* < 0.01.

dsRNA is an intermediate product of viral replication [51]. We investigated the mechanism by which CA inhibits dsRNA synthesis using fluorescence microscopy. We first added PRRSV infected cells for 2 h, discarded the virus venom, added CA, and collected samples at 6 hpi and 24 hpi. As demonstrated in Figure 7A,B, we examined the expression of dsRNA during PRRSV replication after the addition of CA to verify whether CA interfered with viral RNA synthesis. The outcomes showed that under normal conditions, the level of dsRNA increased from 6 h and peaked at 24 h. In contrast, dsRNA production was significantly reduced at both 6 h and 24 h after the addition of CA, with frontal CA hindering dsRNA synthesis. In conclusion, we demonstrated that CA effectively inhibited PRRSV replication and release in Marc-145 cells by impeding viral RNA synthesis (Figure 7C).

## 4. Discussion

The emergence of PRRSV has raised considerable concerns due to the substantial economic losses it has inflicted on the global pig farming industry. Despite extensive and comprehensive research conducted over the years on PRRS, the high variability in PRRSV results in vaccines offering limited protection. Consequently, effectively managing PRRS remains a formidable challenge for the global pig industry [15,52,53,54,55,56].

The compound CA has been reported to exhibit a wide range of antiviral activities including the suppression of Coxsackievirus B3 replication via the TLR4-NF-κB signaling pathway and the reduction in adenovirus-induced inflammation by downregulating caspase-8 and caspase-9 expression [40,57]. These findings highlight the potential of CA as a promising natural antiviral agent. In this study, we observed that CA effectively inhibits PRRSV in Marc-145 cells. Our experimental findings confirm the dose-dependent inhibition of PRRSV by CA (Figure 1A–F and Figure 2A–F), with the most significant inhibitory effect observed at a concentration of 160 μM. CA also shows excellent antiviral effects in PAMs (Figure 3). To further investigate the antiviral activity of CA under different treatment modes, we conducted time-of-addition assay experiments for validation. The results reveal a time-dependent inhibition of PRRSV by CA, with optimal effects observed when it was administered either 8 h before or after infection (Figure 4). Since CA shows the best inhibition when co-treated, we doubt whether CA can interact directly with viruses. The results show that CA could directly interact with virions to inactivate PRRSV (Figure 5). Given that CA exhibited inhibitory effects on various stages including viral attachment, internalization, replication, and release, we designed assays to assess its impact on each stage individually. It is worth noting that our research results indicate that CA does block the binding, entry, replication, and release of PRRSV (Figure 6). According to the replication cycle of PRRSV, the translation of initial NSPs and the formation of RTCs are critical steps in the replication process [58,59]. During viral replication, viral replication enzymes and certain cellular proteins aggregate to form RTCs, with dsRNA serving as an intermediate product [60]. Since CA can inhibit PRRSV during replication, we further examined whether CA suppresses dsRNA synthesis to hinder PRRSV replication. The results demonstrate that CA exhibits remarkable inhibitory effects at both 6 h and 24 h (Figure 7A,B). Our study revealed that CA can directly interact with viruses and obstruct PRRSV binding and entry into Marc-145 cells. CA also inhibits PRRSV replication and release by obstructing viral RNA synthesis in Marc-145 cells (Figure 7C).

NSPs play a crucial role in the formation of the replication–transcription complex (RTC), viral RNA synthesis, and viral replication. Studies have highlighted the significance of NSP2, NSP3, NSP5, NSP9, NSP10, and NSP11 in RTC synthesis. Specifically, NSP2 is responsible for cleaving the NSP2-3 junction and activating NSP4 to process other essential NSPs for RTC formation. Additionally, NSP2 facilitates membrane rearrangement related to the RTC and influences DMV formation. Both NSP3 and NSP5 function as transmembrane proteins that interact with NSP2 to modify membranes and form DMVs. Furthermore, NSP9, NSP10, and NSP11 act as essential components of the RTC by acting as RNA polymerase, RNA helicase, and ribonuclease, respectively [61,62,63]. Our findings suggest that CA has the capacity to inhibit the formation of dsRNA. Therefore, it is crucial to ascertain if CA inhibits dsRNA production by specifically targeting and suppressing one or more of specific NSPs, namely NSP2, NSP3, NSP5, NSP9, NSP10, and NSP11. Additionally, it is important to investigate whether CA also exerts inhibitory effects on these specific NSPs. In subsequent experiments, we will further examine the inhibitory effects of CA on the NSPs that comprise the RTC. Moreover, CA possesses antiviral activity through activation of the TLR 4-NF-κB signaling pathway or the suppression of caspase-mediated endogenous and exogenous pro-apoptotic pathways [64,65]. It is essential to explore the applicability of these findings in relation to PRRSV. CA demonstrates significant anti-inflammatory properties [66], potentially through modulation of stimulated macrophage activation via the MAPK signaling pathway [67]. Furthermore, CA has been shown to inhibit interleukin-1 beta (IL-1β) secretion, raising questions about whether its antiviral activity is mediated through the suppression of inflammatory factor release [68]. Such investigations are vital for further elucidating the mechanisms by which CA exerts its effects against PRRSV. With a long history of clinical application as a feed additive in fattening pigs, CA has demonstrated benefits such as improved growth performance, meat quality enhancement, enhanced antioxidant capacity and immune function, and mitigation of post-mortem muscle glycolysis [69]. These findings highlight the clinical advantages of CA, including its safety and ease of administration. Nevertheless, further research is warranted to determine optimal concentration and in vivo effects for potential therapeutic applications. Subsequently, we will investigate the in vivo antiviral properties of CA to gain insights into its inhibitory effects on PRRSV at molecular levels along with associated signaling pathways and innate immune factors contributing to its clinical antiviral efficacy.

We demonstrated that CA has anti-PRRSV activity in vitro. But it is unclear whether CA can treat pigs infected with PRRSV. Although CA as a feed additive has been documented and its safety can be assured, the dose to control replication of PRRSV in vivo is uncertain. In conclusion, CA still shows great potential as an anti-PRRSV drug.

In conclusion, our research demonstrated that CA could inhibit essential stages of the PRRSV lifecycle: binding, entry, replication, and release. CA could directly interact with PRRSV. Additionally, CA disrupted the expression of dsRNA during viral replication, thereby suppressing in vitro PRRSV replication in Marc-145 cells. As a promising novel antiviral agent, CA exhibits considerable potential for preventing and controlling PRRSV, thus providing new prospects for therapeutic interventions targeting this PRRSV.

## Data Availability

The data presented in this study are available on request from the corresponding author.
